# An integrative review of resilience among nursing students in the context of nursing education

**DOI:** 10.1002/nop2.1559

**Published:** 2022-12-23

**Authors:** Pimwalunn Aryuwat, Margareta Asp, Annica Lövenmark, Matanee Radabutr, Jessica Holmgren

**Affiliations:** ^1^ Mälardalen University Västerås Sweden; ^2^ Praboromarajchanok Institute, Boromarajonani College of Nursing Changwat Nonthaburi The Ministry of Public Health Nonthaburi Thailand

**Keywords:** health, integrative review, nursing education, nursing students, resilience

## Abstract

**Aim:**

This integrative review aimed to examine empirical research on resilience among nursing students in the context of nursing education. Resilience helps nursing students handle challenges, such as changing learning styles and experiencing their first clinical practice.

**Design:**

An integrative review.

**Methods:**

The search terms focused on resilience and health in nursing students and nursing education. The database used in this review were CINAHL Plus, PubMed and MEDLINE. The Mixed Methods Appraisal Tool appraised the studies' quality.

**Results:**

This study explored 52 records and revealed three current research focuses related to nursing students' resilience: (1) the concept and description of resilience, (2) the characteristics affecting resilience and (3) the mediating role of resilience in maintaining holistic health. Recommendations include adding a resilience topic to the nursing curriculum, providing resilience enhancement programs, examining the relationship between resilience and holistic health and exploring the influence of resilience about global health crises.

**Public Contribution:**

Resilience among nursing students plays a vital role in helping them to overcome adversities during their nursing education. Additionally, after graduation, nursing students can continue contributing to society as resilient Registered Nurses in the future.

## 1 INTRODUCTION

Health is precious, as it is preferable for one's state of being and a personal resource to function in everyday life. Health can be viewed positively as the personal ability to accomplish vital goals to make one's life more satisfactory (Nordenfelt, 2014), which is closely aligned with the concept of resilience. Early studies on resilience explored the notion as a trait concept (Genet & Siemer, 2011; Waugh et al., 2011), whereas later studies described resilience as a process deployed in response to complex and challenging events and circumstances (Graham, 2018; Phillips et al., 2019; Schönfeld et al., 2017). Resilience is commonly described as the ability to bounce back from negative challenges and adversities in life (Anderson et al., 2019; Baltacı, 2021). In addition, the concept of resilience as a process of adaptation is associated with what Nordenfelt (2014) has described as the second‐order ability of a person to achieve their vital goals in the given or acceptable circumstances.

## 2 BACKGROUND

Transitioning from adolescence to adulthood is a challenging part of life for nursing students as they have to face new and unexpected challenges, such as increased use of digital tools in the classroom and clinical education and changing timetable of clinical practice (Agu et al., 2021; Aslan & Pekince, 2021; Huang et al., 2020; Sveinsdóttir et al., 2021). Nursing students have to deal with new experiences both in the classroom and in clinical education, such as being challenged by new pedagogy and taking care of real patients for the first time during the first clinical practice. They must adapt to the higher education system (which sometimes requires that they live away from their families), deal with the high expectations of academic achievement and face new peer pressure challenges (Hollenbach, 2016; Olsen, 2018; Patton et al., 2016; Shearer, 2016; Simpson & Sawatzky, 2020). During their education, more complex challenges may ascend, such as uncertainty of course deadlines, extended duration of clinical practice, being under a high level of stress and lockdown of their institutes during new emerging pandemics (Agu et al., 2021; Harries et al., 2021; Huang et al., 2020; Luberto et al., 2020, Roldán‐Merino et al., 2022; Usher et al., 2020).

Resilience is broadly recognized as one of the vital facets related to one's positive health (Phillips et al., 2019; Santrock, 2019). A high level of resilience is advantageous for adolescents when they face the challenges and responsibilities of adulthood, especially when experiencing adverse circumstances (Graham, 2018; Phillips et al., 2019; Schönfeld et al., 2017). Several challenging problems may arise during the transition period for nursing students. First, the transition periods from adolescence to adulthood and from academic learning to clinical training may put them under a lot of pressure that can put their health at risk, especially their mental health (Phillips et al., 2019; Santrock, 2019). Furthermore, resilience has been recognized as essential for undergraduate and graduate nursing students (Jackson, 2018). Recent studies in nursing education have indicated the importance of resilience as a positive influence that can help nursing students deal with the adversities, such as the impact on their quality of life, increased level of academic burnout and psychological distress during the lockdown period of the severe pandemic (Cuartero & Tur, 2021; Guillasper et al., 2021; Labrague & Ballad, 2021; Sweeney, 2021; Usher et al., 2020).

Numerous studies have suggested that resilience could contribute to personal growth due to the experience of negative, challenging and difficult circumstances throughout one's academic years (Amsrud et al., 2019; Kaewmanee et al., 2018; Olsen, 2018; Sangon et al., 2018). However, most studies on resilience and health in nursing students have mainly focused on the relationship between resilience and psychological health or well‐being (Alzayyat & Al‐Gamal, 2014; Delgado et al., 2017; Miremadi, 2015; Patton et al., 2016). Thus, a need to explore the relationship between resilience and a more holistic understanding of health. In addition, understanding how resilience in nursing students is conceptualized in the context of academic learning and, more specifically, in clinical training would be valuable. Further research is also needed in this regard (Reyes et al., 2015a; Thomas & Revell, 2016).

Two previous reviews related to resilience in nursing students provide key information on the general concept of resilience and some factors that can affect it in nursing students, especially in the context of clinical education Reyes et al., 2015a; Thomas & Revell, 2016). However, the definitions and concepts of resilience have changed over time and are commonly clarified according to specific populations (Stephens, 2013). Although there is a growing amount of research on resilience in nursing, a clear explanation of resilience in nursing students remains limited (Reyes et al., 2015b). Empirical studies on resilience have led researchers in the nursing field to examine the phenomenon in the nursing discipline, especially in the context of new and critical challenges that have emerged in nursing education, such as the recent trend of nursing studies on the effects of the COVID‐19 pandemic (Agu et al., 2021; Cygan et al., 2021). The variation in definitions and conceptual descriptions of resilience in nursing and nursing education may create confusion or ambiguity for researchers. Therefore, the concept of resilience needs to be clarified in the context of nursing education.

This integrative review aimed to investigate empirical research on resilience among nursing students in the context of nursing education. The purpose of the integrative review was to answer the following research questions:

1. How is resilience among nursing students conceptualized in the context of nursing education in empirical research?
2. What may impact resilience in nursing students in the context of nursing education?
3. How is resilience promoted in nursing students in the context of nursing education?

## 3 DESIGN

In this study, Whittemore and Knafl's framework of integrative review method was used to analyze and synthesize both quantitative and qualitative publications about resilience in the context of nursing students (Whittemore & Knaft, 2005). The integrative review method allowed for the consideration of empirical research related to resilience in nursing education. Whittemore and Knafl's framework for data collection, analysis and synthesis consists of five stages: problem identification, literature search, data evaluation, data analysis and presentation.

## 4 METHOD

### 4.1 Literature search

The literature search was conducted using the following databases: CINAHL Plus, PubMed and MEDLINE, with support from a librarian at *Mälardalen University*. The keywords used in the search included the terms resilience, resiliency and health, along with the following terms: nursing students, student nurses, nursing education, clinical practice and clinical education. Publications were included if (a) the findings addressed resilience in nursing students, (b) participants in the studies were nursing students, (c) the study design was empirical, (d) the content was written in English and (e) the date of publication was between January 2011 and December 2021.

The reasons for choosing publications after January 2011 were to isolate the most recent literature addressing the research questions and to review the empirical studies that best reflected the recent issues related to resilience in nursing education. Publications were excluded if (a) they focused on resilience among other populations, such as other healthcare students, new nurse graduates, school nurses and nurse educators; (b) they discussed resilience as an implication rather than part of the study findings; (c) they were not empirical research studies, such as discussion and review papers; (d) they were unpublished dissertations and theses; (e) they could not be accessed in full‐text form and (f) they did not focus on nursing education.

**FIGURE 1:**
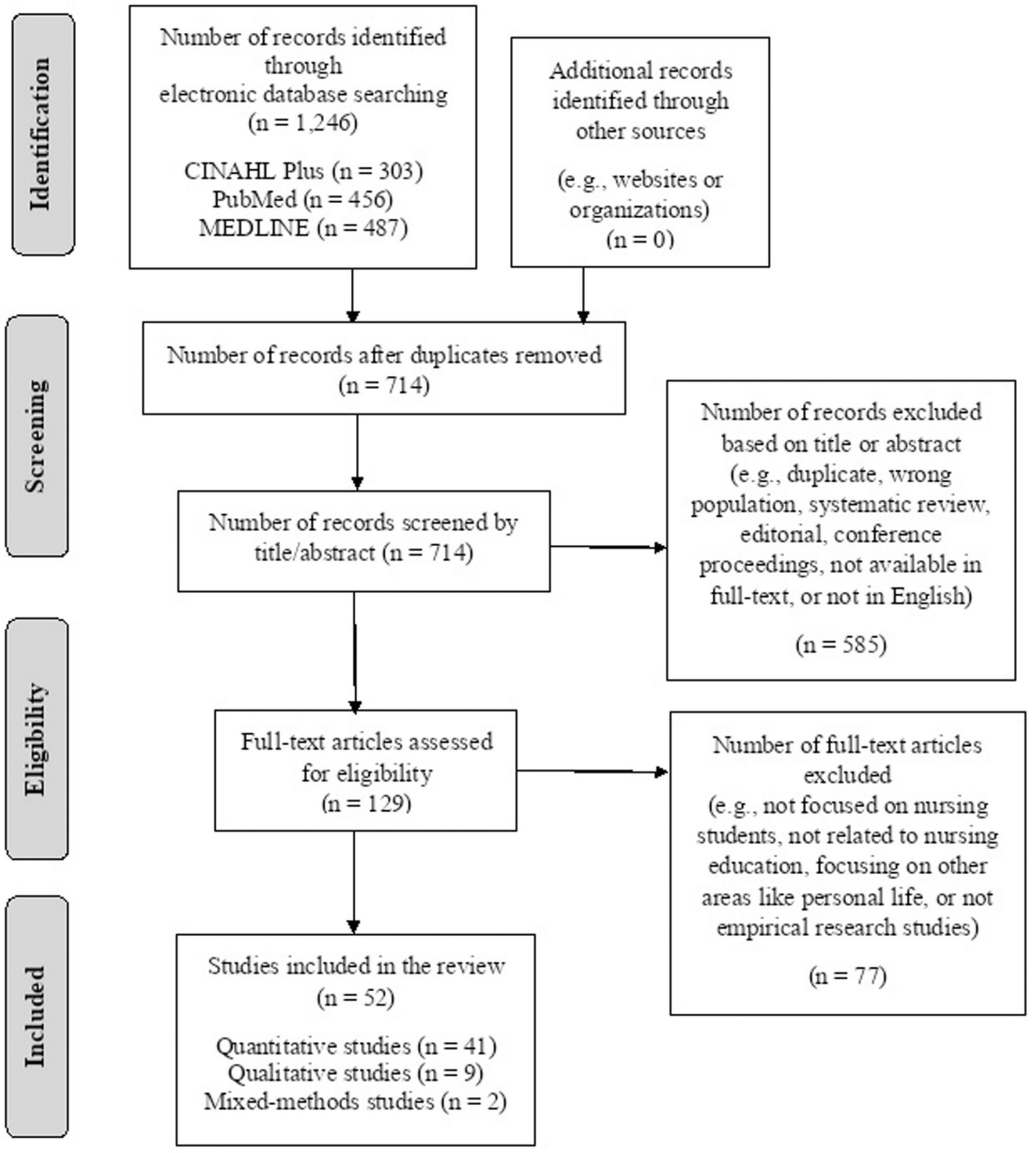
PRISMA flow diagram of the literature search

The search strategy was carried out, documented and illustrated, as shown in Figure 1 (PRISMA flow diagram of the literature search, available to view in the online version), based on the Preferred Reporting Items for Systematic Reviews and Meta‐Analyses (PRISMA) guidelines (Moher et al., 2009). A total of 1246 records were retrieved. Additional records identified through other sources (e.g., websites and organizations) were not included in this integrative review.

The topics of the 1246 records were primarily reviewed to screen for duplication of the studies, after which some duplicate articles were removed. A total of 714 abstracts were reviewed to search for relevance based on the inclusion criteria. Next, 585 records were excluded for not focusing on resilience in nursing students, not being original research articles, or addressing resilience as an implication or intervention rather than as part of the study findings; hence, these were excluded from the review. The remaining 129 records were assessed for eligibility, of which 77 were excluded for reasons such as not focusing on nursing students, not being related to nursing education and not being empirical research studies. Finally, 52 publications consisting of 41 quantitative studies, nine qualitative studies and two mixed methods studies met the inclusion criteria and were included in the data analysis of this integrative review.

### 4.2 Data evaluation

Records included in the final sample were appraised for quality using the Mixed Methods Appraisal Tool (MMAT; Hong et al., 2018). The MMAT is a critical tool for the appraisal stage of reviews that includes qualitative, quantitative and mixed methods studies. The tool assesses the methodological quality of five types of studies: qualitative research, randomized controlled trials, non‐randomized studies, quantitative descriptive studies and mixed methods studies (Hong et al., 2018). The 52 included studies were evaluated for quality based on the MMAT checklist. The characteristics and summaries of the included studies are illustrated in Table 1 (Characteristics and summary of included studies, available to view in the online version).

**TABLE 1:** Characteristics and summary of included studies

No.	Author/Year/Country	Aim	Sample	Method	Instruments	Findings	Implications
1	Abiola et al. (2017 ) Nigeria	To examine the elements of well‐being that led to resilience among undergraduate nursing students	71 undergraduate nursing students	Cross‐sectional design	Resilience Scale (RS)/Self‐Scoring PERMA Scale/demographic form	Resilience has a positive relationship with an individual's well‐being. Nursing students with higher resilience were identified as having higher levels of well‐being. Positive emotions and engagement were two elements of well‐being that significantly differentiated nursing students with different resilience levels	Future research on developing a program related to resilience and the sub‐elements of well‐being was recommended
2	Arries‐Kleyenstüber (2021) Canada	To explore self‐reported resilience about ethical ideology among undergraduate nursing students, compare differences in scores and examine the relationships between the study variables and selected demographic characteristics	80 undergraduate nursing students	Cross‐sectional design	Resilience Scale^TM^/Ethics Position Questionnaire (EPQ)/demographic form	Age and year of study had no relationship with resilience among nursing students. Only gender was associated with resilience, with male nursing students scoring lower on resilience and equanimity than female nursing students. Moreover, personal values and interpersonal factors may explain the different levels of resilience in different genders	Faculty members need to pay attention to the influences of gender on perceiving resilience among nursing students. The findings of this study can provide support and strategies for nursing students to cope with stressors based on gender differences
3	Beauvais et al. (2014) United States	To describe the relationship between emotional intelligence, psychological empowerment, resilience, spiritual well‐being and academic success in undergraduate and graduate nursing students	73 undergraduate nursing students/51 graduate nursing students (total *N* = 124)	Cross‐sectional design	Spreitzer Psychological Empowerment Scale/Wagnild and Young Resilience Scale/Spiritual Well‐Being Scale/Mayer–Salovey–Caruso Emotional Intelligence Test/demographic form	Resilience, psychological empowerment and spiritual well‐being positively affected academic success among graduate nursing students but not undergraduate nursing students	This study pointed out the implications of exploring the factors that can help increase academic success in undergraduate nursing education
4	Ching and Cheung (2021) China	To gather data from students in pre‐registration healthcare disciplines to identify the factors that influence their resilience	1070 undergraduate nursing students/133 medical laboratory science students/65 radiography students/52 optometry students (total *N* = 1320)	Cross‐sectional design	Connor–Davidson Resilience Scale (CD‐RISC)/General Self‐Efficacy Scale (GSE)/Cognitive and Affective Mindfulness Scale, Revised Cognitive and Affective Mindfulness Scale (CAMS‐R)/Positive and Negative Affect Scale (PANAS)/Brief Cope Scale (BCS)/demographic form	Age was associated with all students' resilience, though some factors affected resilience among students from different disciplines. Among nursing students, gender and financial assistance were associated with resilience, whereas the year of study was indicated as significant among medical laboratory science students and having a paid job was reported among optometry students. In addition, all nine predictors in the study affected resilience among nursing students	Nursing instructors and clinical preceptors should work together to enhance resilience in nursing students and other healthcare students. The enhancement of resilience could help them with personal growth and professional development
5	Ching et al. (2020) China	To explore the stressors and coping patterns of nursing students with differing levels of resilience and burnout during clinical placements	24 undergraduate nursing students	Qualitative descriptive design	Semi‐structured interview	Two main themes were revealed: (1) stressors arising from the students aligning their expectations with the demands of the clinical placement and (2) coping as a process of fitting into the ward culture. High resilience and low burnout levels among nursing students were associated with self‐directed goals and coping by using self‐regulation strategies. In contrast, low resilience and high burnout were related to external orientation and self‐blame strategies	The authors recommended three approaches to help nursing students during their clinical practice: (1) offering interventions to empower nursing students to fit into the clinical environment, (2) inspiring engagement in self‐reflection and (3) encouraging the use of both personal and external resources
6	Chow et al. (2018) China	To examine the relationship between resilience and well‐being among university nursing students in Hong Kong	678 undergraduate nursing students	Cross‐sectional design	Connor–Davidson Resilience Scale (CD‐RISC)/World Health Organization‐5 Well‐Being Index (WHO‐5)/demographic form	Resilience had a positive effect on perceived well‐being among both undergraduate and postgraduate nursing students. In addition, postgraduate nursing students had higher levels of resilience than undergraduate nursing students	Nursing educators and stakeholders should focus on educational strategies to enhance resilience levels among nursing students in the nursing curriculum. Moreover, developing a supportive learning environment that can foster resilience in nursing students is crucial
7	Chow et al. (2020) China	To develop a resilience‐building module for university nursing students and evaluate its effects on resilience, well‐being and mindfulness	195 undergraduate nursing students	Mixed‐methods design	Connor–Davidson Resilience Scale (CD‐RISC)/World Health Organization‐5 Well‐Being Index (WHO‐5)/Mindfulness Attention Awareness Scale (MAAS)/focus group interviews/demographic form	Mindfulness was associated with resilience. Additionally, nursing students were pleased with the learning experience of the resilience‐building module, which evoked their awareness of resilience	Including the resilience‐building module in the undergraduate nursing, curricula could be beneficial. Moreover, better mindfulness was acknowledged as vital for enhancing resilience among undergraduate nursing students
8	Clohessy et al. (2019) Australia	To gain insight into how midwifery students conceptualize resilience and explore how education might support its development	Six undergraduate midwifery students	Concept analysis design	Focus group/thematic analysis	Four main themes were identified: (1) resilience is triggered by exposure to adversity, (2) resilience consists of being able to bounce back, (3) resilience enables you to move forward and (4) resilience is important for midwifery students	Crucial strategies were recommended for enhancing resilience among undergraduate midwifery students, such as building self‐confidence, inspiring optimism and providing social support
9	Devi et al. (2021) Indonesia	To reveal whether resilience mediates the associations among stress, depression and anxiety	336 undergraduate nursing students	Cross‐sectional design	Connor–Davidson Resilience Scale (CD‐RISC)/Depression, Anxiety and Stress Questionnaire (DASS‐42)/demographic form	Stress and resilience had a negative relationship. Nursing students were found to be vulnerable to psychological health problems, such as anxiety and depression, during clinical practice. Additionally, stress was one of the critical predictors of anxiety and depression among nursing students, especially during clinical practice. Resilience was identified as a mediator between stress and both anxiety and depression. This study found that nursing students with high levels of resilience were at a low risk of anxiety and depression during their clinical practice	As resilience is a crucial mediator between clinical practice and stress, depression and anxiety levels among nursing students, strategies to enhance resilience in this population should be explored
10	Dong et al. (2021) China	To analyze how family resilience mediates the relationship between childhood trauma and psychological resilience among undergraduate nursing students	698 undergraduate nursing students	Cross‐sectional design	Childhood Trauma Questionnaire (CTQ)/Family Resilience Assessment Scale (FRAS)/Connor–Davidson Resilience Scale (CD‐RISC)/demographic form	Nursing students' psychological resilience was associated with childhood trauma and family resilience. Those who had experienced childhood trauma reported having low psychological resilience. Family resilience was confirmed not only to be a mediator between childhood trauma and psychological resilience but also to have a negative relationship with childhood trauma. Moreover, family resilience positively correlated with psychological resilience among nursing students	Promoting family interventions may be advantageous to the enhancement of resilience in nursing students, especially among those who have experienced childhood trauma
11	Drach‐Zahavy et al. (2021) Israel	To examine nursing students' stress and ability to cope with the coronavirus disease 2019 (COVID‐19) pandemic through an ecological model of resilience	492 undergraduate nursing students	Cross‐sectional design	NASA Task Load Index/Coping Strategies Inventory Short Form (CSISF)/Connor–Davidson Resilience Scale (CD‐RISC)/Preparedness Scale/Trust in Administrators Scale/Strain Symptoms Questionnaire (SSQ‐14)/demographic form	Resilience decreases the level of stress in nursing students. Strain symptoms were negatively associated with trait resilience, perceptions of positive responses from the university regarding the pandemic and having trust in national policymakers. In contrast, disengagement‐in‐emotion coping strategies were positively related to strain symptoms	Resilience in nursing students can be developed and influenced through academic learning and clinical training
12	Eaves and Payne (2019) United Kingdom	To explore the relationship between perceived stress, resilience, burnout and the intention to leave midwifery among midwifery students	150 undergraduate midwifery students	Cross‐sectional design	Perceived Stress Scale (PSS)/Oldenburg Burnout Inventory (OLBI)/Resilience Scale (RS)/intention to quit questions/demographic form	The findings revealed a relationship between resilience, stress and burnout. Stress predicted disengagement and emotional exhaustion was predicted by stress and year of study. A high level of stress and a low level of resilience predicted the intention to quit a midwifery program. Resilience was indicated as an essential factor to help reduce the choice to quit. Moreover, resilience did not affect burnout among students but helped preserve their interest in the profession and prevented them from leaving their education	Resilience may be helpful for nursing students to continue with their education while they are working in the nursing profession
13	Elzohary et al. (2017) Egypt	To determine the relationship between levels of ego resilience, perceived stress and degree of life satisfaction among faculty nursing students at Damanhour University	520 undergraduate nursing students	Cross‐sectional design	Connor–Davidson Resilience Scale (CD‐RISC)/Perceived Stress Scale (PSS)/Satisfaction with Life Scale (SWLS)/demographic form	Ego resilience had a negative relationship with perceived stress but a positive relationship with life satisfaction in nursing students. This indicated that a high level of ego resilience might reduce perceived stress in nursing students and increase their life satisfaction. In addition, predictive factors related to high ego resilience among nursing students were participating in academic/social activities, social support, year of enrollment and a higher level of life satisfaction. In contrast, a high level of perceived stress predicted low ego resilience	Nursing educators should pay more attention to developing and improving resilience levels in nursing students, which can benefit graduates with high academic and clinical competence in their efforts to adjust to work adversities
14	Ertekin Pinar et al. (2018) Turkey	To investigate midwife candidates' psychological resilience, self‐confidence and problem‐solving skills	270 undergraduate midwifery students	Cross‐sectional design	Psychological Resilience Scale for Adults (PRSA)/Self‐Confidence Scale (SCS)/Problem Solving Inventory (PSI)/demographic form	The findings showed a positive relationship between psychological resilience, self‐confidence and problem‐solving skills. Age was associated with self‐confidence and psychological resilience, whereas living area was associated with self‐confidence and problem‐solving skills. In addition, perceived social support was related to psychological resilience and problem‐solving skills and monthly income was related to psychological resilience	Preventive programs regarding factors affecting students' resilience, self‐confidence and problem‐solving skills were recommended. Furthermore, future studies that examine low levels of resilience, self‐confidence and problem‐solving skills based on different years of study should be considered
15	Fernández‐Martínez et al. (2021) Spain	To analyze how students' levels of resilience and fear of death evolve in the first three years of the degree and whether there are differences between students based on age and gender and to describe the relationship between resilience and fear of death	100 undergraduate nursing students	A comparative, correlational and longitudinal study	Collet–Lester Fear of Death Scale (CLFODS)/Resilience Scale (ER‐14)/demographic form	Fear of death among nursing students was reported at medium to high levels throughout all academic years and was associated with their age. In addition, the study year was related to nursing students' resilience levels	Although this study revealed that fear of death was not related to resilience among nursing students, the authors emphasized the importance of resilience in clinical education for nursing students
16	Froneman et al. (2016) South Africa	To explore and describe nursing students' views on the essential elements required for an effective educator–student relationship to strengthen their resilience and the educator–student relationship	40 undergraduate nursing students	Explorative, descriptive and contextual qualitative design	World Café Method/content analysis	The findings revealed five main themes; (1) teaching–learning environment, (2) educator–student interaction, (3) educator qualities, (4) staying resilient and (5) strategies to strengthen resilience. Nursing students needed a caring and supportive environment, constructive interaction, acknowledged human rights and suitable non‐verbal communication. The crucial characteristics of nursing educators are binarizing, caring, respectful, responsible, moral, patient, open‐minded, motivation and punctual	Nursing educators should focus on creating a more positive and effective educator–student relationship and practice strategies to strengthen nursing students' resilience. Moreover, nursing students must be informed about the importance of resilience
17	García‐Izquierdo et al. (2018) Spain	To analyze the role of resilience in the dimensions of academic burnout syndrome and psychological health in a sample of nursing students	218 undergraduate nursing students	Cross‐sectional design	Connor–Davidson Resilience Scale (CD‐RISC)/Maslach Burnout Inventory Student Survey (MBI‐SS)/General Health Questionnaire (GHQ‐12)/demographic form	The findings showed an association between burnout, emotional exhaustion and self‐efficacy with resilience and psychological health in nursing students. In addition, the study revealed the moderating role of resilience in psychological health in cases of emotional exhaustion	The measurement of appropriate training for nursing students was recommended. Moreover, future research investigating the relationship between resilience and academic burnout and the effect of resilience on psychological health is crucial
18	Grande et al. (2021) Saudi Arabia	To investigate the relationship between nursing students' profile variables and their mental well‐being and resilience during the coronavirus disease‐19 (COVID‐19) pandemic and how this impacts their understanding of holistic nursing care provision	579 undergraduate nursing students	Cross‐sectional design	Connor–Davidson Resilience Scale (CD‐RISC)/Warwick–Edinburgh Mental Well‐Being Scale (WEMWBS)/demographic form	There were no significant differences in resilience level based on age, gender and year of study. However, nursing students' academic performance affected their resilience, such that nursing students with higher grade point averages (GPA) were reported to have higher resilience levels. Moreover, a positive relationship between resilience and mental well‐being was reported among nursing students during the pandemic	Nursing students' assessment of resilience and mental well‐being should be carried out through academic support by nursing educators and preceptors
19	Hamadeh Kerbage et al. (2021) Australia	To explore undergraduate nursing students' resilience, challenges experienced and supports utilized during the pandemic	340 undergraduate nursing students	Mixed‐method design	Connor–Davidson Resilience Scale (CD‐RISC)/narrative reflection/demographic Form	Resilience levels were higher in employed nursing students than those unemployed. In addition, resilient nursing students' resilience level is sorted to be higher than those of others. Three main themes were identified: (1) fear of the virus, (2) isolation and (3) mental health problems. Essential coping strategies for nursing students included developing daily routines, staying connected and establishing self‐help techniques	Nursing educators and stakeholders should develop strategies for enhancing nursing students' resilience during their nursing education. Future research to assess the resilience of nursing students after the COVID‐19 pandemic subsides is recommended. Moreover, supporting strategies for the nursing curriculum were suggested to benefit nursing education
20	Hasson et al. (2021) United Kingdom	To test the differences in resilience, stress and well‐being and explore the correlations between nursing students in China and those in the United Kingdom	444 undergraduate nursing students	Cross‐sectional design	Connor–Davidson Resilience Scale (CD‐RISC)/WHO 5 Well‐Being Index/ Perceived Stress Scale (PSS)/demographic form	Age and intention to leave among nursing students in both countries affected resilience. Moreover, psychological well‐being was associated with resilience and stress level, such that stress and resilience were identified as affecting factors and resilience was a protective factor	Future research should examine interventions and coping strategies that help younger nursing students cope with stress during their nursing education. In addition, a more extensive cohort study of educational institutions and nursing students' year groups is recommended. Moreover, policymakers in nursing education should develop support mechanisms and policies to help nursing students reduce stress and improve their resilience
21	Hwang and Shin (2018) South Korea	To determine the characteristics of nursing students with high academic resilience	254 undergraduate nursing students	Cross‐sectional design	Resilience Scale (RS)/Clinical Practice Stress Scale/Clinical Practice Satisfaction Scale/demographic form	High levels of academic resilience in nursing students were associated with having good interpersonal relationships, high academic grades, role models and high satisfaction with one's major. Moreover, nursing students with a high academic resilience level were reported to have lower clinical practice stress levels and more increased social‐affective capabilities than those with lower academic stress levels	The findings showed that nursing students with a high level of resilience are less likely to quit their studies and more likely to benefit from activities that support their social‐affective skills. The nursing curriculum should be embedded with programs that can improve the social‐affective competence of nursing students
22	Keener et al. (2021) United States	To examine the relationship between quality of life (QoL), resilience and associated factors among nursing students during the unprecedented COVID‐19 pandemic and subsequent social distancing requirements	130 undergraduate nursing students/22 postgraduate nursing students (total *N* = 152)	Cross‐sectional design	WHO Quality of Life‐BREF (WHOQOL‐BREF)/Connor–Davidson Resilience Scale (CD‐RISC)/demographic form	QoL in nursing students was related to resilience, having online experience and being well‐prepared for online learning. This study highlighted resilience as a predictive variable for QoL in nursing students	A hybrid online learning model should be integrated into the nursing curriculum, improving the online learning experience and professional development opportunities. Online resources should be well provided. Moreover, future research to evaluate resilience enhancement programs for nursing students should be carried out
23	Kim (2019) South Korea	To examine the influence of resilience, life satisfaction and psychological well‐being on attitudes toward death	184 undergraduate nursing students	Cross‐sectional design	Interpersonal Reactivity Index (IRI)/Life Satisfaction Scale (LSS)/Psychological Well‐Being Scale (PWB)/Attitude to Death Scale/demographic form	Attitude to death was related to nursing students' resilience, life satisfaction and psychological well‐being. Resilience had a positive relationship with life satisfaction and psychological well‐being, whereas life satisfaction had a positive relationship with the psychological well‐being of nursing students	Death‐related education and end‐of‐life care should be added to the nursing curriculum as they can benefit nursing students in perceiving death as a path of life. This could help them effectively handle such incidents related to death during their nursing education
24	Kim et al. (2021) United States	To explore the influence of coping mechanisms as predictors of stress, anxiety and depression among nursing students during the COVID‐19 lockdown	173 undergraduate nursing students	Cross‐sectional design	Connor–Davidson Resilience Scale (CD‐RISC)/Spirituality Support Scale/Family APGAR Questionnaire /Perceived Stress Scale (PSS)/General Anxiety Disorder‐7 (GAD‐7)/Patient Health Questionnaire‐9 (PHQ‐9)/demographic form	A high level of resilience and family functioning were related to a lower risk of stress, anxiety and depression among nursing students during the COVID‐19 lockdown. Also, a lower risk of depression was associated with high levels of spiritual support	A resilience‐building program for nursing students should be developed and integrated into the nursing curriculum and implemented through lectures, reflective journaling and assignments. Such a program could benefit students with stress management regarding academic, social and personal diversity. In addition, resilience interventions and training to improve the psychological well‐being of nursing students are essential
25	Kong et al. (2016) China	To examine the positive association between emotional intelligence and clinical communication ability among practice nursing students and determine whether resilience moderates the relationship between emotional intelligence and clinical communication ability among Chinese nursing students	377 undergraduate nursing students	Cross‐sectional design	Emotional Intelligence Inventory (EII)/Connor–Davidson Resilience Scale (CD‐RISC)/Clinical Communication Ability Scale (CCAS)/demographic form	The findings showed a positive relationship between emotional intelligence and clinical communication ability among nursing students. Specifically, resilience was identified as a factor that moderated this relationship	Developing resilience intervention strategies is recommended, as they could help enhance nursing students' clinical communication abilities and emotional intelligence
26	Kotera et al. (2021) United Kingdom	To appraise relationships of self‐compassion through experiences of resilience, engagement, motivation and well‐being in UK nursing students	182 undergraduate nursing students	Cross‐sectional design	Self‐Compassion Scale‐Short Form (SCS‐SF)/Brief Resilience Scale (BRS)/Utrecht Work Engagement Scale for Students (UWES‐S)/Academic Motivation Scale (AMS)/Short Warwick–Edinburgh Mental Well‐Being Scale (SWEMWBS)/demographic Form	While self‐compassion had a positive relationship with resilience, engagement, intrinsic motivation and mental well‐being, it had a negative relationship with motivation. In addition, resilience and mental well‐being were essential predictors of self‐compassion	Nursing educators and preceptors can help nursing students foster self‐compassion through resilience training and mental well‐being practices
27	Labrague (2021) Philippines	To examine the direct and indirect effects of stress associated with the pandemic on student nurses' life satisfaction and psychological well‐being through the intermediary role of resilience	301 undergraduate nursing students	Cross‐sectional design	Perceived Stress Scale (PSS)/Psychological Well‐Being Scale (PWBS)/Brief Multidimensional Students' Life Satisfaction Scale (BMSLSS)/Brief Resilient Coping Scale (BRCS)/demographic Form	During the COVID‐19 pandemic, resilience was reported to reduce the negative effects of pandemic‐associated stress on nursing students' life satisfaction and psychological well‐being	Enhancing resilience can help sustain mental and psychological well‐being and expand life satisfaction in nursing students
28	Lee and Kim (2021) South Korea	To examine the mediating effects of emotional intelligence and resilience on the relationship between type D personality and caring ability in nursing students	278 undergraduate nursing students	Cross‐sectional design	Type D Scale‐14 (DS14)/Wong and Law Emotional Intelligence Scale (WLEIS)/Connor–Davidson Resilience Scale (CD‐RISC)/demographic form	Type D personality was associated with emotional intelligence, resilience and caring ability. Emotional intelligence was identified as a mediator between type D personality and caring ability	An evaluation of emotional intelligence in nursing students should be included in an intervention to improve the students' caring abilities. Also, promoting nursing students' emotional intelligence strategies needs to be implemented
29	Lekan et al. (2018) United States	To explore resilience in senior‐level baccalaureate nursing students	100 undergraduate nursing students	Cross‐sectional design	Connor–Davidson Resilience Scale (CD‐RISC)/one open‐ended question about the experience of resilience/demographic form	Resilience in nursing students was associated with academic stressors and support resources. The authors recommended that nursing educators help nursing students develop resilience, which will benefit nursing students regarding their academic success and the smooth transition into professional nursing roles in the future	Nursing students with complex family and employment commitments may be vulnerable to impaired resilience. Future studies to further explore resilience in this population may benefit nursing education
30	Li et al. (2014) China	To investigate the relationships among post‐traumatic growth, emotional intelligence and psychological resilience in vocational school nursing students who had experienced childhood adversities	202 undergraduate nursing students	Cross‐sectional design	Childhood Adversities Checklist (Chinese version)/Posttraumatic Growth Inventory/Emotional Intelligence Scale/Connor–Davidson Resilience Scale (CD‐RISC)/demographic form	Post‐traumatic growth had a positive relationship with emotional intelligence and psychological resilience	This study addressed that resilience and emotional intelligence can help nursing students deal with difficulties in their clinical practices
31	Liang et al. (2019) Taiwan	To develop and implement a resilience enhancement (RE)‐based project for Taiwanese nursing students during their Last Mile practicum	28 undergraduate nursing students	Participatory action research (PAR) design	Group discussion/individual interviews/reflective diaries	The findings revealed three main themes: (1) increasing self‐exploration, (2) furthering confidence and competency and (3) constructing resilience. In addition, improving nursing knowledge and skills and practicing positive thinking and behavior can enhance resilience in nursing students	The findings of this study can be used as a guideline to design resilience enhancement programs for nursing students during clinical education. Such programs would provide psychological support and lead to the improvement of professional competence
32	Lopez et al. (2018) Singapore	To understand how undergraduate nursing students perceive and experience their clinical placements and to identify the factors that helped them build resilience	19 undergraduate nursing students	Qualitative content analysis design	Focus group interviews	The findings revealed two major categories: (1) challenges faced during clinical placements and (2) building resilience over time. During their first clinical placement, nursing students experienced stress because of inadequate peer and clinical support. Resilience developed over time and helped them adapt to the culture of clinical placements through peer support	Nursing students' resilience can be developed through clinical education. Resilience programs related to coping strategies and mindfulness training should be integrated into the nursing curriculum
33	Mathad et al. (2017) India	To identify the correlates and predictors of resilience among nursing students	194 undergraduate nursing students	Cross‐sectional design	Freiburg Mindfulness Inventory (FMI)/Connor–Davidson Resilience Scale (CD‐RISC)/Toronto Empathy Questionnaire (TEQ)/Perseverative Thinking Questionnaire (PTQ)/demographic form	Nursing students' resilience was associated with mindfulness, perseverative thinking and empathy	This study addressed the importance of resilience and mindfulness in nursing education
34	Mcdermott et al. (2020) USA	To test a positive psychology‐oriented model detailing the potential links between (a) psychological resilience, (b) depressive symptoms, (c) intrapersonal well‐being, (d) interpersonal well‐being and (e) academic distress in nursing students.	933 undergraduate nursing students	Cross‐sectional design	Brief Resilience Scale (BRS)/Patient Health Questionnaire‐9 (PHQ9)/Brief Inventory of Thriving (BIT)/Healthy Minds Study (HMS)/demographic Form	Decreasing depression levels enhanced resilience levels in nursing students. This study highlighted that resilience is critical to the academic well‐being of nursing students, especially within the unsupportive contexts of the nursing students' mental health	Nursing educators and policymakers should consider contextual factors, such as providing a positive campus climate. Moreover, resilience training programs can benefit nursing students' academic success and mental well‐being
35	Mott (2014) USA	To examine the lived experiences of undergraduate nursing students with faculty bullying	Six undergraduate nursing students	Phenomenological design	In‐depth individual interviews	The findings revealed four main themes: (1) emotional experiences of bullying, (2) giving and gaining mutual respect, (3) value of resilience and persistence and (4) perception is reality. Resilience and persistence are crucial factors for nursing students to overcome bullying. Resilient behaviors were identified as not wanting to quit or adjusting to oneself	Nursing educators' ability to engage in positive interactions with students should be evaluated. Moreover, a workshop on positive interactions with students should be provided for nursing educators
36	Orkaizagirre‐Gómara et al. (2020) Spain	To develop a predictive model for perceived competence and to obtain an integrator evaluation of the nursing curriculum with measures of nursing self‐efficacy, general self‐efficacy, resilience and stress among nursing students	265 undergraduate nursing students	Cross‐sectional design	The Nursing Clinical Skills Self‐Efficacy Scale (CSES)/Generalized Self‐Efficacy Scale (GSE)/Brief Resilience Scale (BRS)/Perceived Competence for Nursing Students (PCNS)/Kezkak‐Questionnaire on Stressors/demographic form	Perceived competence in nursing students was predicted by nursing self‐efficacy, general self‐efficacy, resilience and the year of the degree course	An integrated evaluation model can help predict clinical performance in nursing students. Future research related to resilience, self‐efficacy, perceived competence and stress should be carried out within the context of clinical practice. Moreover, comparative studies in different clinical contexts and cultures are recommended
37	Ozsaban et al. (2019) Turkey	To evaluate the levels of psychological resilience, academic stress and social support available to nursing students and the relationship between these factors	322 undergraduate nursing students	Cross‐sectional design	Psychological Resilience Scale for Adults (PRSA)/Nursing Education Stress Scale (NESS)/Multidimensional Scale of Perceived Social Support (MSPSS)/demographic form	A positive relationship between resilience and perceived social support in nursing students was addressed. Resilience was affected by the individual characteristics of nursing students. Additionally, this study showed no relationship between resilience and academic stress	Future studies are recommended to understand better the factors affecting nursing students' resilience and how they can be improved. Better theoretical models related to resilience among nursing students should be developed and evaluated
38	Reyes et al. (2015) Canada	To explore nursing students' understanding and enactment of resilience	38 undergraduate nursing students	Grounded theory design	In‐depth individual interviews	The basic social process associated with the understanding and enactment of resilience in nursing students was identified as “pushing through,” which they employed to handle adversities during their education. Resilience can be learned and developed, as it is viewed as a dynamic and contextual process instead of a static trait or personality characteristic	Future research focusing on theory development within the context of different types of nursing education programs and diverse cultural backgrounds is recommended. In addition, the findings of this study addressed the need to integrate the topic of nursing students' resilience into the nursing curriculum
39	Ríos‐Risquez et al. (2016) Spain	To examine the relationship between resilience, academic burnout and psychological health in a sample of nursing students	113 undergraduate nursing students	Cross‐sectional design	Connor–Davidson Resilience Scale (CD‐RISC)/Maslach Burnout Inventory Student Survey (MBI‐SS)/General Health Questionnaire (GHQ‐12)/demographic form	Relationships among resilience, emotional exhaustion and psychological health were reported. The resilience and emotional exhaustion predicted perceived psychological health. Moreover, resilience was related to psychological discomfort and academic burnout	Developing resilience in nursing students is important and integrating resilience into nursing education is essential
40	Sahu et al. (2019) India	To explore the relationship between levels of stress and resilience with the use of mobile phones in nursing students	72 undergraduate nursing students/30 postgraduate nursing students (Total *N* = 102)	Cross‐sectional design	Perceived Stress Scale (PSS)/Connor–Davidson Resilience Scale (CD‐RISC)/Mobile Phone Involvement Questionnaire (MPIQ)/demographic form	The findings showed a relationship between perceived stress and both resilience and age. The capacity to recover was higher in graduate nursing students than undergraduate students. In addition, a relationship between high mobile phone use and perceived stress was found	Stress management and life skills training programs are crucial. A psycho‐educational module or intervention to support nursing students' resilience, enhance their stress management skills and promote the healthy use of mobile phones is recommended
41	Sam and Lee (2020) India	To assess the perceived stress and resilience levels of nursing students	620 undergraduate nursing students	Cross‐sectional design	Resilience Scale (RS)/Perceived Stress Scale (PSS)/demographic form	Severe stress and low resilience levels in nursing students were reported, as well as a relationship between perceived stress and resilience	Assessing nursing students' resilience is essential and the topic of resilience should be embedded in the nursing education program
42	Serçe et al. (2021) Turkey	To determine the predictive role of nursing students' characteristics and psychological resilience in psychological distress	652 undergraduate nursing students	Cross‐sectional design	General Health Questionnaire (GHQ‐12)/Resilience Scale (RS)/demographic form	Psychological distress in nursing students was affected by suffering from a chronic disease, being a woman, poor academic achievement, choosing the profession so as not to be unemployed, being single and having a low resilience level	It is recommended that psychological distress be assessed among nursing students and an intervention be developed to enhance resilience in at‐risk nursing students
43	Sigalit et al. (2017) Israel	To explore (a) the associations between students' personal and group resilience, their utilization of social networking platforms and formally taught communication skills; (b) students' personal and clinical characteristics related to personal and group resilience and the perceived helpfulness of a communication course; and (c) factors that contribute to students' satisfaction with their clinical placement	149 undergraduate nursing students	Cross‐sectional design	Connor–Davidson Resilience Scale (CD‐RISC)/social media use questionnaire/one open question on satisfaction with clinical placement/demographic form	The findings showed a relationship between social media use and nursing students' resilience. Additionally, social media use, religion and clinical placement characteristics were related to the communication course's resilience and perceived helpfulness	Social media use can be used to promote resilience among nursing students
44	Smith and Yang (2017) China	To examine the relationship between stress and resilience on psychological well‐being in a cohort of Chinese undergraduate student nurses	1538 undergraduate nursing students	Cross‐sectional design	Stress in Student Nursing (SINS‐CN)/Resilience Scale (RSCN)/General Health Questionnaire (GHQ 12)/demographic form	Relationships between resilience and stress and psychological well‐being in nursing students were found	Resilience training programs and effective coping strategies among nursing students should be developed to help them handle adversities. The authors recommended that resilience training and effective coping strategies should be promoted among nursing students
45	Spurr et al. (2021) Canada	To examine resilience and wellness, together with the key factors that promote well‐being, in nursing students from a mid‐western Canadian university	196 undergraduate nursing students	Cross‐sectional design	Health Status Survey/Professional Quality of Life (ProQOL) Survey/Cantril Well‐Being Scale/Ego‐Resilience Scale (ER89)/demographic form	There was a relationship between wellness and resilience in nursing students	Understanding how resilience and wellness are influenced throughout the nursing program can be the key to developing targeted wellness initiatives for nursing students
46	Tian et al. (2019) China	To explore the status and influencing factors of vertical workplace violence among Chinese nursing students and to examine the relationship between resilience and such violence	486 undergraduate nursing students	Cross‐sectional design	Resilience Scale of University Students (RSUS)/Questionnaire on Nursing Students' Workplace Vertical Violence/demographic form	Nursing students commonly experience vertical workplace violence during clinical practices. This study pointed out that resilience is a positive factor that helps them deal with these issues	Future study related to exploring and expanding the role of resilience within the context of clinical practice is recommended
47	Van Hoek et al. (2019) Belgium	To explore the influence of sociodemographic factors, resilience and stress‐reducing activities on academic outcomes in undergraduate nursing students, including the intention to leave, academic success and dropout rates	554 undergraduate nursing students	Cross‐sectional design	VK+ Resilience Scale/P3 Palliative Behavior Scale/demographic form	High levels of resilience in nursing students predicted their academic success, intentions to leave and dropout rates. Sociodemographic factors were not associated with educational outcomes	A future cohort study focusing in depth on dropout rates among undergraduate nursing students is recommended
48	Vestphal et al. (2020) Denmark	To investigate the lived experiences of undergoing a nursing education as an emotionally insecure student	Seven undergraduate nursing students	Phenomenological‐hermeneutic design	Semi‐structured interview	Having insecure emotions was linked to feeling shame in nursing students	Nursing educators must be aware of potential misunderstandings and avoid shaming nursing students. Reflection sessions can help nursing students handle emotionally charged experiences. It is crucial to help nursing students explore protective factors and resilience, as these can be their hidden resources for managing feelings of emotional insecurity
49	Wang et al. (2021) Taiwan	To explore the academic resilience of undergraduate nursing students during their Adulting Nursing practicums and identify protective factors to mitigate their impact	19 undergraduate nursing students	Grounded theory design	Individual in‐depth interviews	The main challenges during the clinical practicum of nursing students were facilitators, the environment and the application of techniques. Additionally, academic resilience can be defined as the ability to uphold optimism while dealing with clinical challenges. The crucial outcome of an educational resilience development process is overcoming ignorance through clinical practice	It is recommended that nursing educators and preceptors provide suitable teaching strategies and academic support to help nursing students improve their resilience
50	Yıldırım et al. (2021) Turkey	To investigate the effects of communication skills on resilience in undergraduate nursing students in Turkey	687 undergraduate nursing students	Cross‐sectional design	Resilience Scale for Adults (RSA)/Communication Skills Scale (CSS)/demographic form	Resilience in nursing students was associated with some sociodemographic variables, including marital status, year of study, income, living conditions, social support, self‐coping mechanisms and perceived health status. In addition, resilience was affected by personal communication skills	Interventions regarding communication skills and factors affecting nursing students' resilience should be implemented
51	Yıldız (2021) Turkey	To determine the relationship between post‐traumatic growth (PTG), psychological flexibility and psychological resilience in nursing students after the COVID‐19 alarm status	292 undergraduate nursing students	Cross‐sectional design	Acceptance and Action Questionnaire‐II (AAQ‐II)/Brief Resilience Scale (BRS)/Posttraumatic Growth Inventory (PTGI)/demographic form	Resilience in nursing students was related to both post‐traumatic growth and psychological flexibility. In addition, a higher resilience level was found in a group of nursing students with a middle‐income status and stable attitudes toward the profession	Including resilience and psychological flexibility in nursing, the curriculum was suggested. Practical methods, such as reflective practice, problem‐based learning and experiential learning, were recommended as learning and teaching approaches to enhance resilience. Moreover, proper training in areas such as conflict management skills, stress management skills and communication skills should be provided for nursing students
52	Yun et al. (2020) South Korea	To examine the relationships between nursing students' academic motivation, resilience and post‐traumatic growth	291 undergraduate nursing students	Cross‐sectional design	Post‐Traumatic Growth scale (PTG)/Resilience Scale (RS)/academic motivation questions/demographic form	The effects of academic motivation on resilience and post‐traumatic growth in nursing students were found. Additionally, resilience was identified as a mediator between academic inspiration and post‐traumatic growth	A program enhancing post‐traumatic growth through reinforcing intrinsic motivation and resilience should be developed and integrated into the nursing curriculum. Future research should explore resilient nursing students' characteristics and the relationship between academic motivation and nursing retention rates

### 4.3 Data analysis

The final studies selected for the review were empirical in nature. The literature included a variety of study designs, such as descriptive, correlational, longitudinal, mixed methods, participatory action research, grounded theory and phenomenological. Generally, evaluation is more complex with various methodologies (Whittemore & Knafl, 2005). The research questions guided the analysis of the 52 articles, and the studies were compared and analysed for similarities and differences. Data with similar contexts were assembled. The four steps of data analysis, including data reduction, data display, data comparison and conclusion drawing and verification, were applied to obtain mutual themes and patterns and ensure the findings' rigour.

## 5 ETHICS

Research Ethics Committee approval was not required for this study.

## 6 RESULTS

### 6.1 General characteristics of the included studies

The 52 included studies were from 19 countries, including Australia, Belgium, Canada, China, Denmark, Egypt, India, Indonesia, Israel, Nigeria, Philippines, Saudi Arabia, Singapore, Spain, South Africa, South Korea, Taiwan, Turkey, the United Kingdom and the United States. Concerning the participant characteristics in the included records, 48 studies targeted undergraduate nursing students only, with a total of 14,701 participants; three studies targeted both undergraduate and postgraduate levels, and one study targeted both nursing students and other health students from different disciplines. However, the majority of participants in these four studies were undergraduate nursing students, with a total of 1345 participants. The number of postgraduate nursing students was 103, and 250 students from other disciplines were included as participants. In summary, 16,046 undergraduate nursing students participated in the included studies.

### 6.2 Concepts and descriptions of resilience in nursing students

Resilience has been viewed as an ability or process and a psychological trait. Among most included studies, concepts and descriptions of resilience in nursing students were broadly defined with existing concepts and definitions of resilience. Only three studies specifically provided definitions of resilience, while 13 articles did not mention a specific definition. Froneman et al. (2016) defined resilience as the ability of both nursing educators and students to effectively cope with stressors throughout their academic years. In contrast, Keener et al. (2021) referred to resilience among nursing students as a developing process among individuals through individual protective factors to deal with perceived stress and difficulties. Similarly, Reyes et al. (2015b) identified resilience as a dynamic process that allows one to handle adversities and challenges during nursing education.

The majority of the included studies referred to nursing students' resilience as the ability to positively recover or bounce back from facing adversities or opposing challenges (Ching et al., 2020; Clohessy et al., 2019; Drach‐Zahavy et al., 2021; Eaves & Payne, 2019; Ertekin Pinar et al., 2018; Fernández‐Martínez et al., 2021; Grande et al., 2021; Hamadeh Kerbage et al., 2021; Keener et al., 2021; Kim, 2019; Labrague, 2021; Lekan et al., 2018; Liang et al., 2019; Lopez et al., 2018; Mathad et al., 2017; Orkaizagirre‐Gómara et al., 2020; Ozsaban et al., 2019; Sahu et al., 2019; Sam & Lee, 2020; Smith & Yang, 2017; Vestphal et al., 2020; Wang et al., 2021; Yıldırım et al., 2021). Several studies specifically mention resilience in nursing students as the ability to successfully handle academic stressors and attain academic success (Abiola et al., 2017; Beauvais et al., 2014; Chow et al., 2018; Chow et al., 2020; Hwang & Shin, 2018; Ozsaban et al., 2019; Wang et al., 2021).

Additionally, resilience was addressed as contributing to health conditions and well‐being in nursing students (Chow et al., 2018; Eaves & Payne, 2019; Elzohary et al., 2017; Hamadeh Kerbage et al., 2021; Ríos‐Risquez et al., 2016; Serçe et al., 2021 and Spurr et al., 2021). Moreover, resilience was connected to the personal growth of nursing students, as they can learn from overcoming adversities and gain a positive outlook for their future (Hasson et al., 2021; Liang et al., 2019; Lopez et al., 2018). Among the included studies, resilience was described as an important trait or essential characteristic of nursing students. Resilience was described as a fixed trait (Clohessy et al., 2019), a personal resource (Drach‐Zahavy et al., 2021; Yun et al., 2020), a flexible trait (Eaves & Payne, 2019; Grande et al., 2021), a positive adaptation (Ertekin Pinar et al., 2018), psychosocial characteristic or quality (Lee & Kim, 2021; Yıldırım et al., 2021), essential quality (Smith & Yang, 2017), adjustable phenomenon or state (Tian et al., 2019) and a personality trait (Van Hoek et al., 2019) in the included studies.

### 6.3 Characteristics affecting resilience in nursing students

Some demographic characteristics have been shown to affect resilience in nursing students, including inadequate income (Ching & Cheung, 2021; Ertekin Pinar et al., 2018; Hamadeh Kerbage et al., 2021; Yıldırım et al., 2021; Yıldız, 2021), age (Ching & Cheung, 2021; Ertekin Pinar et al., 2018; Hasson et al., 2021), gender (Arries‐Kleyenstüber, 2021; Ching & Cheung, 2021), marital status (Yıldırım et al., 2021), year of study (Elzohary et al., 2017; Fernández‐Martínez et al., 2021; Yıldırım et al., 2021), religion (Sigalit et al., 2017) and living conditions (Yıldırım et al., 2021). However, some have argued that age does not affect resilience (Arries‐Kleyenstüber, 2021; Grande et al., 2021). For instance, Grande et al. (2021) argued that gender and year of study were not associated with resilience in nursing students.

Some intrapersonal characteristics were associated with nursing students' resilience. Specifically, several factors were reported to have positive effects on resilience, including high self‐confidence (Clohessy et al., 2019; Ertekin Pinar et al., 2018), no intention to quit nursing education (Elzohary et al., 2017; Hwang & Shin, 2018), positive thinking (Clohessy et al., 2019; Liang et al., 2019), high self‐efficacy (Ching & Cheung, 2021; García‐Izquierdo et al., 2018), no experience of childhood trauma (Dong et al., 2021), being satisfied with life (Elzohary et al., 2017), an accomplishment of academic success such as having high‐grade point average (GPA) (Grande et al., 2021) and high self‐compassion (Kotera et al., 2021).

Referring to the interpersonal characteristics reported in the included literature, social support was identified in eight studies as an essential factor that can affect nursing students' resilience, with students who received social support being reported to have higher resilience levels (Clohessy et al., 2019; Dong et al., 2021; Elzohary et al., 2017; Ertekin Pinar et al., 2018; Hwang & Shin, 2018; Lekan et al., 2018; Ozsaban et al., 2019; Yıldırım et al., 2021). In addition, mindfulness was another positive influencing factor for developing resilience in nursing students (Ching & Cheung, 2021; Chow et al., 2018; Mathad et al., 2017). Some studies have highlighted that resilience can also be affected by and developed through academic learning, clinical practice and skills training (Ertekin Pinar et al., 2018; Hasson et al., 2021; Liang et al., 2019).

### 6.4 Mediating role of resilience in nursing students' health

Resilience has played a vital role as a mediator between the various factors influencing nursing students' health and well‐being. Devi et al. (2021) indicated that resilience is a vital mediator of psychological health and factors such as stress, anxiety and depression in the context of experiencing clinical practice. Kong et al. (2016) identified resilience as an important mediator between emotional intelligence and clinical communication ability in nursing students during clinical training. During the COVID‐19 pandemic, another study showed that resilience mediated between life satisfaction and well‐being among nursing students (Labrague, 2021).

Resilience can not only be affected by various factors, as described in the previous section, but it can also influence nursing students' health and education. For instance, several studies have shown that nursing students' well‐being is affected by resilience (Abiola et al., 2017; Chow et al., 2018; Hasson et al., 2021; Kim, 2019; Kim et al., 2021; Labrague, 2021; Ríos‐Risquez et al., 2016; Serçe et al., 2021; Smith & Yang, 2017; Spurr et al., 2021). Perceived stress was another factor shown to be affected by resilience among nursing students during their nursing education (Drach‐Zahavy et al., 2021; Elzohary et al., 2017; Kim et al., 2021; Lekan et al., 2018; Sahu et al., 2019; Sam & Lee, 2020; Smith & Yang, 2017).

Additionally, resilience can affect factors that influence nursing students' lives and their contributions to their nursing studies. Life satisfaction was shown to be affected by resilience in studies by Elzohary et al. (2017), Kim (2019) and Labrague (2021). A low level of resilience is associated with high academic stress in others (Mcdermott et al., 2020; Ozsaban et al., 2019; Van Hoek et al., 2019). Moreover, a high resilience level was reported to affect several factors in nursing students, including higher satisfaction and achievement in clinical practice (Lopez et al., 2018); less or no intention to drop out (Eaves & Payne, 2019; Van Hoek et al., 2019); having post‐traumatic growth (Li et al., 2014; Yıldız, 2021) and overcoming bullying during their nursing education (Mott, 2014).

## 7 DISCUSSION

This integrative review revealed that the current scope of knowledge related to resilience among nursing students in the context of nursing education focuses on three main aspects: (1) the concept and description of resilience as either an ability, a process, or a psychological trait; (2) the characteristics affecting nursing students' resilience and (3) the important role of resilience as a mediator in helping nursing students maintain their holistic health.

The concept of resilience among nursing students has been generally mentioned in most literature as a psychological notion. Although nursing students' resilience was mentioned as being associated with nursing students' health, such an understanding of the concept has not yet been clearly described in the context of nursing education. Furthermore, a model with a specific definition of resilience has not been employed and tested in the resilience literature (Stephens, 2013), and a specific description of resilience related to nursing education should be clearly defined in the literature (Thomas & Revell, 2016).

Resilience among nursing students with diverse demographic backgrounds in terms of age, gender and living conditions was found to differ. Specifically, this integrative review revealed that older nursing students seemed to be more resilient. This could reflect the influence of learning and life experiences. Edwards et al. (2016) argued that age could predict resilience only in young students in their study on the predictors of resilience in students between 16–21 years old. Moreover, gender and living conditions were unrelated to students' resilience in other health disciplines, such as medical students (Oliveira et al., 2017). Another study reported that male nursing students have lower resilience during clinical practice, which could be associated with the experience of specific barriers to being a male nursing student, both in school and in social life (Yang et al., 2017). Furthermore, resilience is related to a specific type of personality in nursing students (Škodová & Bánovčinová, 2018).

Intrapersonal characteristics in nursing students, such as high self‐efficacy, being positive, high self‐confidence, high self‐compassion and being satisfied with life, were identified as factors affecting resilience. In response to changes in health care and nursing education related to the advent of the COVID‐19 pandemic, nursing students needed the ability to adapt to new challenges in both classroom‐based and clinical‐based education (Bradford, 2021). In addition, a recent study highlighted the positive relationship between academic self‐efficacy and resilience among nursing students (El‐Sayed et al., 2021; Warshawski, 2022). Traditional classroom activities had to be converted to virtual and distance learning, and nursing practicum rotations had to be managed with limitations. These challenges in nursing education pushed nursing students to develop strong, positive intrapersonal characteristics that could help them increase their resilience.

This integrative review also addressed some vital interpersonal characteristics, such as social support, which play an essential role in maintaining and enhancing nursing students' resilience. During the COVID‐19 pandemic, several studies indicated the importance of the direct effect of social support on nursing students' resilience (Cuartero & Tur, 2021; El‐Sayed et al., 2021; Warshawski, 2022), and a mediated role of resilience between social support and quality of life among nursing students (Pineda et al., 2022). Female nursing students were reported to perceive more social support than male nursing students (Cuartero & Tur, 2021). In addition, a high level of resilience in nursing students was reported to be associated with adequate social support (Caton, 2021). Similarly, a high level of resilience was associated with perceived social support among university students (Hamdan‐Mansour & Hamdan‐Mansour, 2014) and adolescents (Hidayat & Nurhayati, 2019). Moreover, social support was a significant predictor of resilience among adolescents (Çiçek, 2021). Students at the higher education level with a high level of resilience were found to be more likely to successfully reach out for support (Md Khalid, 2021).

Nursing students' resilience can be defined as the ability to adapt to difficult situations and obstacles. Resilience can be viewed as the ability to recover quickly from an adverse, challenging circumstance to accomplish vital goals. Nursing students who have high resilience seem to be able to connect with their inner strengths, which helps them bounce back from negative challenges during their nursing education. The COVID‐19 pandemic is one example of negative encounters impacting nursing students' stress levels (Gallego‐Gómez et al., 2020). Therefore, resilience can help nursing students maintain their holistic health and well‐being and cope with stress during their nursing education. Bouncing back can be a valuable means to maintaining one's holistic health (Nordenfelt, 2014). In addition, Ozsaban et al. (2019) pointed out that nursing students with high levels of resilience will be more likely to successfully recover from unpleasant experiences that can affect their physical, mental and social health, which they may encounter in both classrooms and clinical placements. A recent study on resilience in nursing students during the COVID‐19 pandemic defined resilience in nursing students as the ability related to successful adaptation to life‐threatening conditions (Mohammadi et al., 2022).

### 7.1 Limitations

The findings of this review may be limited by the inclusion criteria applied to the studies found in a specific database. The included studies were also limited to those published from 2011–2021. Since only empirical studies on resilience in nursing students written in English were reviewed, it is possible that studies in other languages could have improved our understanding of nursing students' resilience.

## 8 CONCLUSION

This integrative review explored and analysed the scope of knowledge related to resilience among nursing students in the context of nursing education. Three key themes were highlighted, which include the concept and description of resilience, the characteristics of nursing students that affect their resilience and the vital role of resilience as a mediator of holistic health in nursing students. Furthermore, this integrative review has some critical implications for nursing research. First, stakeholders in nursing education should add the topic of resilience to nursing curricula and provide special training to enhance resilience in nursing students. Future research should focus on exploring the connection between resilience and holistic health in nursing students. Moreover, research focusing on the relationship between nursing students' resilience and their demographic, intrapersonal and interpersonal characteristics would be a valuable contribution to nursing education.

The findings of this integrative review have some implications for nursing education. Nursing educators, nursing preceptors and policymakers are encouraged to add the topic of resilience to the nursing curriculum to improve nursing education. In addition, specific programs related to nursing students' intrapersonal characteristics to enhance resilience should be provided as brief training sessions before they begin their clinical education. Furthermore, the interpersonal characteristics of nursing students, such as social support, must be considered throughout their nursing education. This integrative review also suggests that future research on resilience and holistic health among nursing students needs to be conducted. Specifically, more research related to resilience in nursing education should focus on exploring the relationship between resilience and the characteristics that affect its development among nursing students. Furthermore, future research on the influence of resilience in nursing students in the context of global health crises, such as emerging infectious diseases, may significantly contribute to nursing education and the nursing profession.

## AUTHOR CONTRIBUTIONS

PA has assumed overall responsibility for the study. However, MA, AL, MR and JH have contributed to the design and planning of the study, writing of the manuscript, critical discussions and revisions. PA was responsible for collecting, mapping and initially analysing the data. The final analysis of the data and the framing of the results were performed together by all authors. PA revised the final manuscript and completed it for submission.

## FUNDING INFORMATION

The study was funded by Mälardalen University and Praboromarajchanok Institute, the Ministry of Public Health Thailand.

## CONFLICT OF INTEREST

The authors have no conflict of interest to declare.

## Data Availability

Data openly available in a public repository that issues datasets with DOIs.
